# Do both the research community and the general public share an interest in the sleep–pain relationship, and do they influence each other?

**DOI:** 10.3389/fpsyg.2023.1198190

**Published:** 2023-07-21

**Authors:** Tor Arnison, Xiang Zhao

**Affiliations:** ^1^Clinical Epidemiology and Biostatistics, Faculty of Medicine and Health, School of Medical Sciences, Örebro University, Örebro, Sweden; ^2^Unit of Psychology, School of Behavioural, Social and Legal Sciences, Örebro University, Örebro, Sweden

**Keywords:** sleep, pain, published research, public interest, Google searches, Big Data

## Abstract

**Introduction:**

Chronic pain and sleep disturbance bidirectionally influence each other in a negative spiral. Although this academic knowledge is known by researchers, it is imperative to bridge it over to the general public because of its applied implications. However, it is unclear how academia and the general public reciprocally shape each other in terms of knowledge of the sleep–pain relationship. The purpose of this study was (1) to assess the longitudinal trajectories of research on the sleep–pain relationship and the general public’s interest in this topic and (2) to examine whether the academic interest leads to the general public’s interest, or vice versa.

**Methods:**

We used a Big Data approach to gather data from scientific databases and a public search engine. We then transformed these data into time trends, representing the quantity of published research on, and the general public’s interest in, the sleep–pain relationship. The time trends were visually presented and analyzed *via* dynamic structural equation modeling.

**Results:**

The frequency of both published articles and searches soared after 2004. Published research leads to an increased interest in the sleep–pain relationship among the general public but does not predict more published articles. Furthermore, the general public’s interest reinforces itself over time but does not predict published research.

**Conclusion:**

These results are encouraging because it is essential for research on the sleep–pain relationship to reach a broader audience, beyond the walls of academia. However, to prevent a potential alienation between academic and practical knowledge, we encourage openness among researchers to being inspired by the general public’s knowledge of the sleep–pain relationship.

## 1. Introduction

Chronic pain is among the leading causes of disability duration worldwide (James et al., 2018) and is notoriously difficult to treat (Williams et al., 2020). Sleep disturbance and pain bidirectionally influence each other (Andersen et al., 2018), and sleep disturbance has proven to be a more salient predictor of future pain than vice versa (Finan et al., 2013; Arnison et al., 2022; Varallo et al., 2022). Sleep problems in themselves are associated with considerable disability and suffering (Bin et al., 2012; Seng et al., 2016), and chronic pain comorbid with sleep disturbance is associated with particularly severe illness profiles (Burgess et al., 2019; Arnison et al., 2022). It is therefore crucial to increase our understanding of the relationship between pain and sleep problems in order to untangle this negative spiral of disability and suffering.

The silver lining to this relationship is that psychological interventions for sleep problems have been found to be both efficacious and time- and cost-effective (Morgenthaler et al., 2006). The potential carryover effects of sleep interventions on pain problems are promising (Tang, 2018; Selvanathan et al., 2021), in particular because chronic pain is challenging to treat (Williams et al., 2020). Cognitive–behavioral therapy for insomnia, in addition to effectively reducing insomnia in those who suffer from comorbid insomnia and chronic pain, has also been shown to have small to moderate effects on chronic pain (Selvanathan et al., 2021). The crux of the matter is that chronic pain may be reduced or prevented by treating insomnia. This is relevant not only for clinicians but also for the general public, because self-help treatments for sleep problems have also been proven effective (van Straten and Cuijpers, 2009). If the general public were to gain knowledge of the relationship between sleep problems and pain, they may be able to help themselves reduce the suffering from both. To facilitate self-learning, it is imperative that the academic knowledge of the sleep–pain relationship, which is well known by researchers, bridges over to the general public.

Evidence shows that society drives what research topics gain the most attention, and the societal impact of research is an important process that has garnered much interest in recent years because of this (Bornmann, 2012; Fecher and Hebing, 2021). However, little is known about the trend of academic interest in the sleep–pain relationship and the general public’s interest in it. It is also thus far unclear how academia and the general public reciprocally shape each other in terms of knowledge and understanding of the sleep–pain relationship.

In the present study, we first aimed to examine the across-time relationship between academic interest and public interest in the sleep–pain relationship. Our second aim was to examine whether research on the sleep–pain relationship has led to an increased interest of this topic in the general public and whether the general public’s interest in the sleep–pain relationship has led to more research on sleep and pain. The findings from this study could also elucidate the knowledge dissemination from academia to the public and provide insights into health education and communication.

## 2. Methods

### 2.1. Data extraction

The current study was a retrospective, infodemiological project that assessed how research on the sleep–pain relationship and the general public’s interest in the same relationship has changed over time and how the two are related to each other. Data retrievals were conducted in August 2022. All code was written in Python 3.1 *via* the interface Jupyter (Kluyver et al., 2016).

#### 2.1.1. Published scientific articles on the sleep–pain relationship

Most research is published in academic journals, which are indexed in scientific databases. We therefore used published articles on the sleep–pain relationship as a proxy for the research/academic world’s interest in the sleep–pain relationship. To ensure a comprehensive body of research, we collected articles from both PubMed (which is medically oriented) and Scopus (which includes more general science, including social sciences).

#### 2.1.2. Google searches on sleep and pain

Whereas people seek online information *via* multiple searching methods, Google is the most popular search engine with a market share of over 93 percent, as of the beginning of 2023 (StatCounter, 2023). We used the number of Google searches as the proxy for the general public’s interest in the sleep–pain relationship.

To collect Google searches on the sleep–pain relationship over time, we used the pseudo-Application Programming Interface (API) Google Trends and the data wrapper Pytrends (Hogue and Wilde, 2023). We collected a time-series trend of Google searches from 2004 to 2022 (Google data are available from 2004 onward). The criteria for the retrieved Google searches were that they had to contain both the words “sleep” and “pain.”

### 2.2. Data collection

#### 2.2.1. Research articles

We used a Big Data approach to collect the data from both the Scopus API’s (Scopus) and the National Center for Biotechnology Information (NCBI) Entréz API (PubMed). We based our article retrieval, cleaning, and merging procedures of articles on code provided by Santos et al. (2020).

When collecting the data from the Scopus API, we used the data wrapper Pybliometrics. First, we used the ScopusSearch API to search for relevant articles and retrieving a list of their Scopus EIDs. We used the search terms TITLE-ABS-KEY (Insomnia OR Sleep) AND TITLE-ABS-KEY (Pain OR Ache) AND (LIMIT-TO (LANGUAGE, “English”)). Then, for each article we collected the Title, Abstract, Keywords, EID, Digital Object Identifier (DOI), and PubMed ID (PMID), *via* the AbstractRetrieval API, and saved them into a Comma Separated Values (CSV) file. When collecting data from the NCBI Entréz API, we used the data wrapper Pymed to first collect the articles and then save them into a CSV file. We used the same search terms as for the Scopus API. Next, we cleaned the two data sets so that they would be compatible and then merged them into a large data set while removing duplicates.

Next, we used supervised machine learning with binary classification (Müller and Guido, 2017) to sort out the articles concerning the sleep–pain relationship. These articles were coded as the positive class, and others were coded as the negative class. We based our analysis on findings by Bannach-Brown et al. (2019). They established a procedure for conducting systematic reviews using a machine learning algorithm to sort out relevant articles that equaled—or even outperformed—the traditional human procedure, with a 98.7% sensitivity and 86% specificity. On the basis of their findings and recommendations, we chose to base our analysis on a logistic regression bag-of-words model. For each article, we tokenized the texts in the title and the abstract and combined them into a vectorized matrix of token counts. We then used a hyperparameter tuner to identify to identify the optimal parameters for the logistic regression model. For these procedures, we used the Scikit learn library (Pedregosa et al., 2011).

We drew a randomized sample of 500 articles from the final data set, read the titles and abstracts, and categorized these as either being about the sleep–pain relationship or not. Sixty of these 500 (12%) articles were categorized as being in the positive (sleep–pain) class, and 440 of the articles were categorized as being in the negative (other) class. To this sample we then added another 140 articles that we previously knew concerned the sleep–pain relationship. We did this to add information about articles on the sleep–pain relationship and thereby facilitate the algorithm’s ability to identify such articles. We trained our machine learning algorithm on 75% of the sample using a hyperparameter tuner to identify the optimal parameters for the model and subsequently tested it on the remaining 25%, because this ratio has been recommended as a good rule of thumb (Müller and Guido, 2017). The training data was used to evaluate the quality of the algorithm. Finally, we applied the algorithm on the total data set to extract the articles on the sleep–pain relationship.

### 2.3. Data analysis

To be able to analyze the change in published research and google searches over time, and to compare the two types of data, we transformed them into time-series data with frequency of published articles or google searches per month and normalized the frequencies from 0 (minimum frequency) to 100 (maximum frequency). First, we analyzed each time-trend separately and visually. Then, we combined them and compared them visually. When comparing the time-trends, we limited them from 2004 to 2022, since Google data is only available from 2004.

To analyze how the time-trends related to each other, we used dynamic structural equation modeling (DSEM). This is a novel analysis method, using Bayesian estimation that allows for accounting of autoregressive and cross-lagged effects in data with few cases and many measurement points (Asparouhov et al., 2018). There is often considerable dependency between adjacent timepoints in time-series data, and not accounting for these autoregressive effects may severely bias the results (Geiser, 2021). We first used DSEM to assess how the two time trends correlated, first through a simple correlation and then while controlling for autoregressive effects in both variables. We then added bidirectional, cross-lagged effects of published articles and Google searches (maintaining the autoregressive effects in each time trend), to examine the longitudinal, reciprocal relationship between the time trends.

## 3. Results

### 3.1. Data collection

The data retrievals were conducted in August 2022. The article search on Scopus yielded 45,408 retrieved articles, and the search on PubMed yielded 20,873. Of these articles, 14,385 were removed as duplicates because they were retrieved from both Scopus and PubMed, resulting in a final raw sample of 51,896 articles.

The machine learning algorithm that was identified as optimal in extracting articles on the relationship between sleep and pain was a logistic regression model using unigram “bag-of-words,” “stop words.” On the test sample, the model showed a good F1 score of 0.82 of selecting articles on the sleep–pain relationship, and a very good F1 score of 0.91 in excluding articles that concerned both sleep and pain, but not the relationship between them. With regard to selecting articles on the sleep–pain relationship, the sensitivity was 0.83, and the specificity was 0.91. Out of the total 51,896 articles, the machine learning model identified 4,357 as concerning the sleep–pain relationship, representing 8.4% of the total sample. These results correspond well to the results by Santos et al. (2020; see Methods section), although the sensitivity and inclusion rates were lower. This may mean that the current study slightly underestimated the number of articles on the sleep–pain relationship that have been published.

Google searches on sleep and pain were retrieved from January 2004 through August 2022. The frequency of searches per month were normalized from 0 (lowest) to 100 (highest) throughout the measured period.

### 3.2. Published research articles on the sleep–pain relationship over time

In total, 4,357 articles on the sleep–pain relationship were identified, ranging from September 1896 to August 2022. The first identified article was written by Patrick and Gilbert and published in *The Psychological Review* in September 1896. The article described a series of experiments on the effects of sleep loss on, among other aspects, pain sensitivity. However, the authors did not draw any specific conclusions on the effects of sleep deprivation on pain sensitivity (Patrick and Gilbert, 1896).

**Figure 1:**
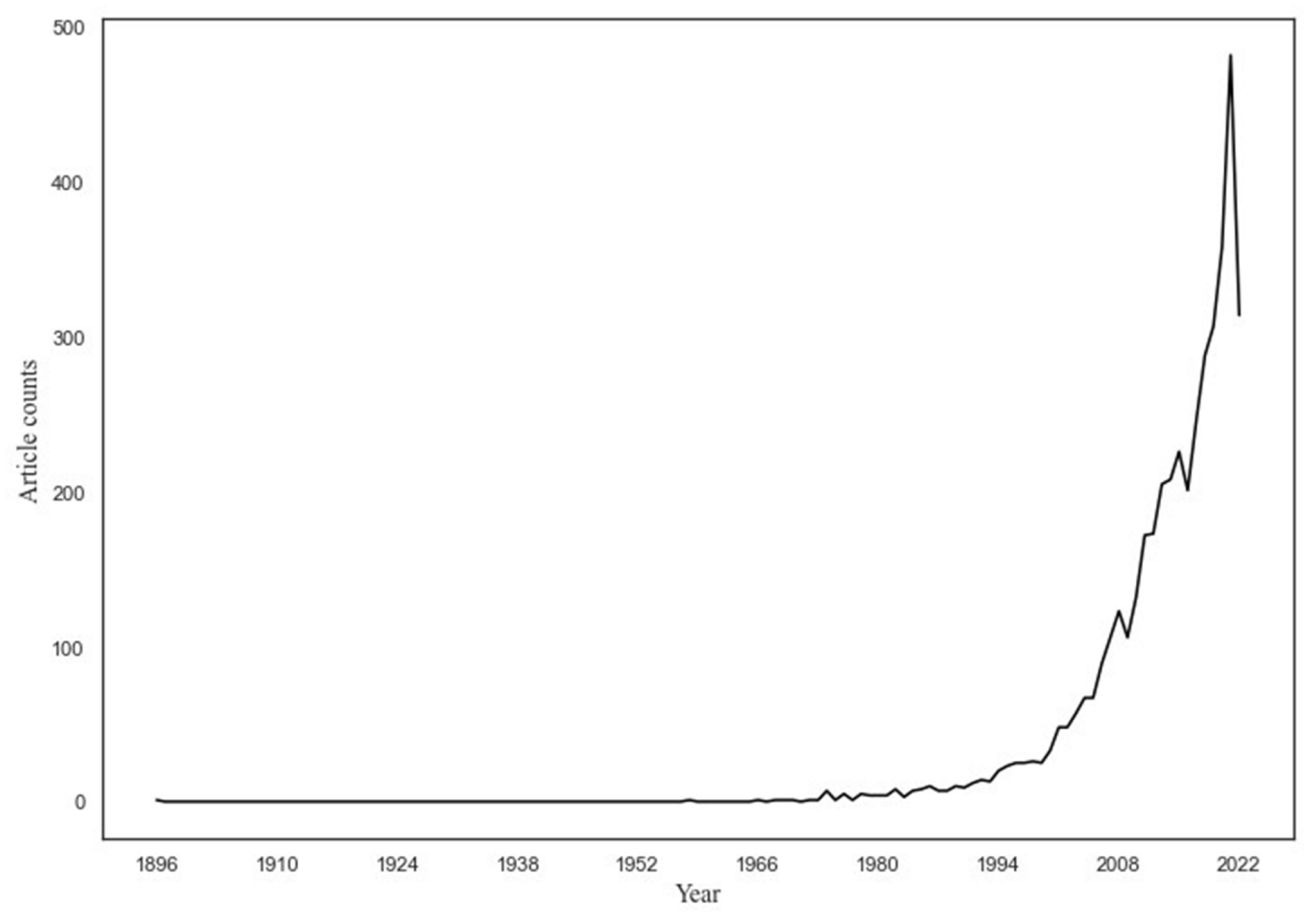
Depiction of numbers of published articles on the sleep–pain relationship, from 1896 to 2022. Published articles in years is depicted on the *x*-axis, and frequency of articles per year is depicted on the *y*-axis. To facilitate readability, the *x*-axis is depicted in years instead of months.

As can be seen in Figure 1, very few articles on the sleep–pain relationship were published before 1960. In the following 40 years, the frequency of published articles increased slightly. After the year 2000, however, the frequency of published articles increased exponentially. The trend dropped in the end, because 2022 was only two thirds through at the time of data retrieval.

### 3.3. Google searches on sleep and pain over time

**Figure 2:**
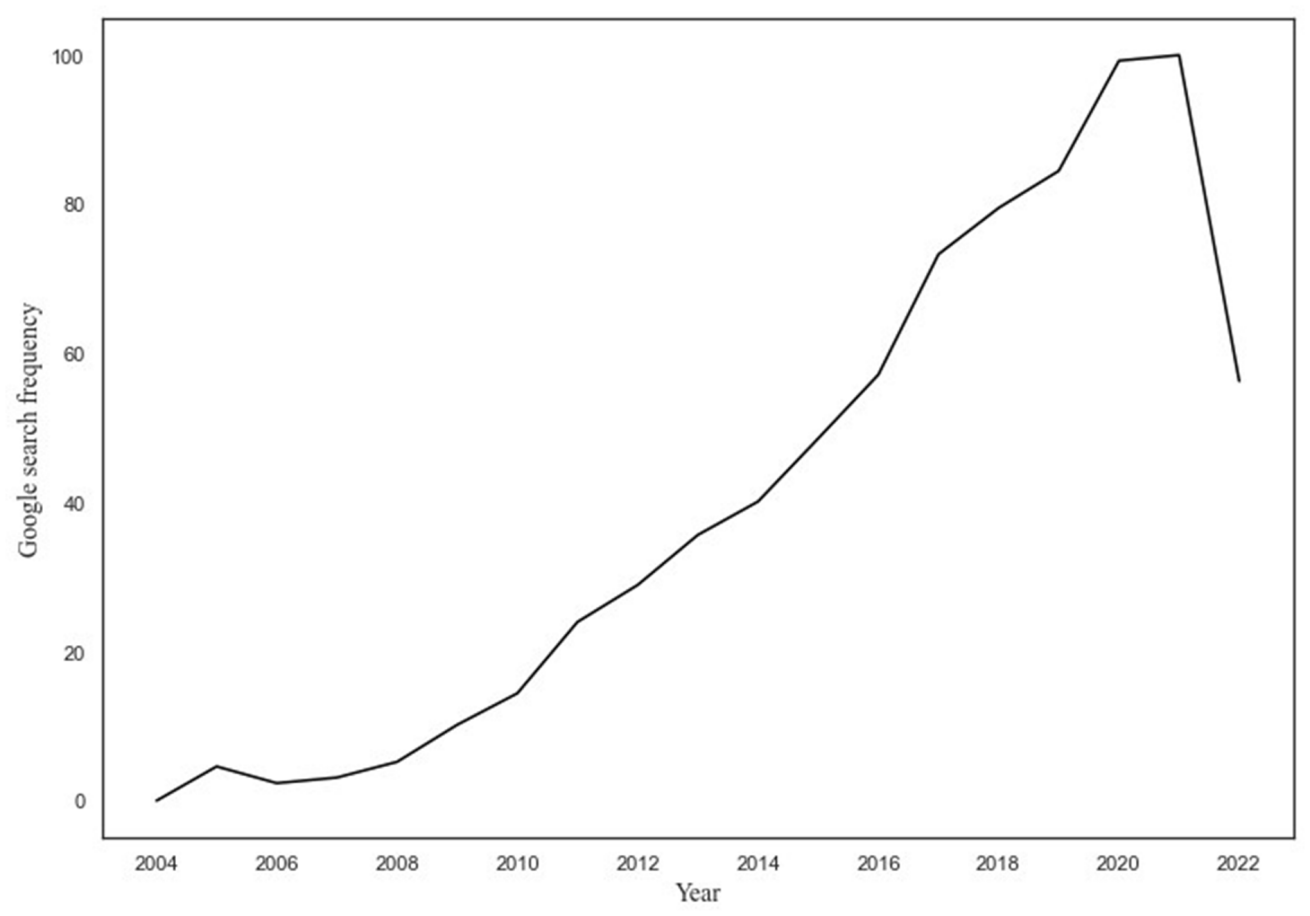
Depiction of the change in Google searches on sleep and pain from 2004 to 2022. Number of searches per year is depicted on the *x*-axis, and frequency of Google searches is depicted on the *y*-axis. To facilitate readability, the *x*-axis is depicted in years instead of months.

Data on Google searches were collected from 2004 until August 2022. As can be seen in Figure 2, the number of searches concerning sleep and pain steadily increased over the course of these years, with the fewest searches in 2004. The trend peaked in 2020 and 2021 and thereafter appeared to drop. However, this is probably due to the fact that 2022 was not over at the time of the data collection.

### 3.4. Association between published articles on the sleep–pain relationship with Google searches on sleep and pain

**Figure 3:**
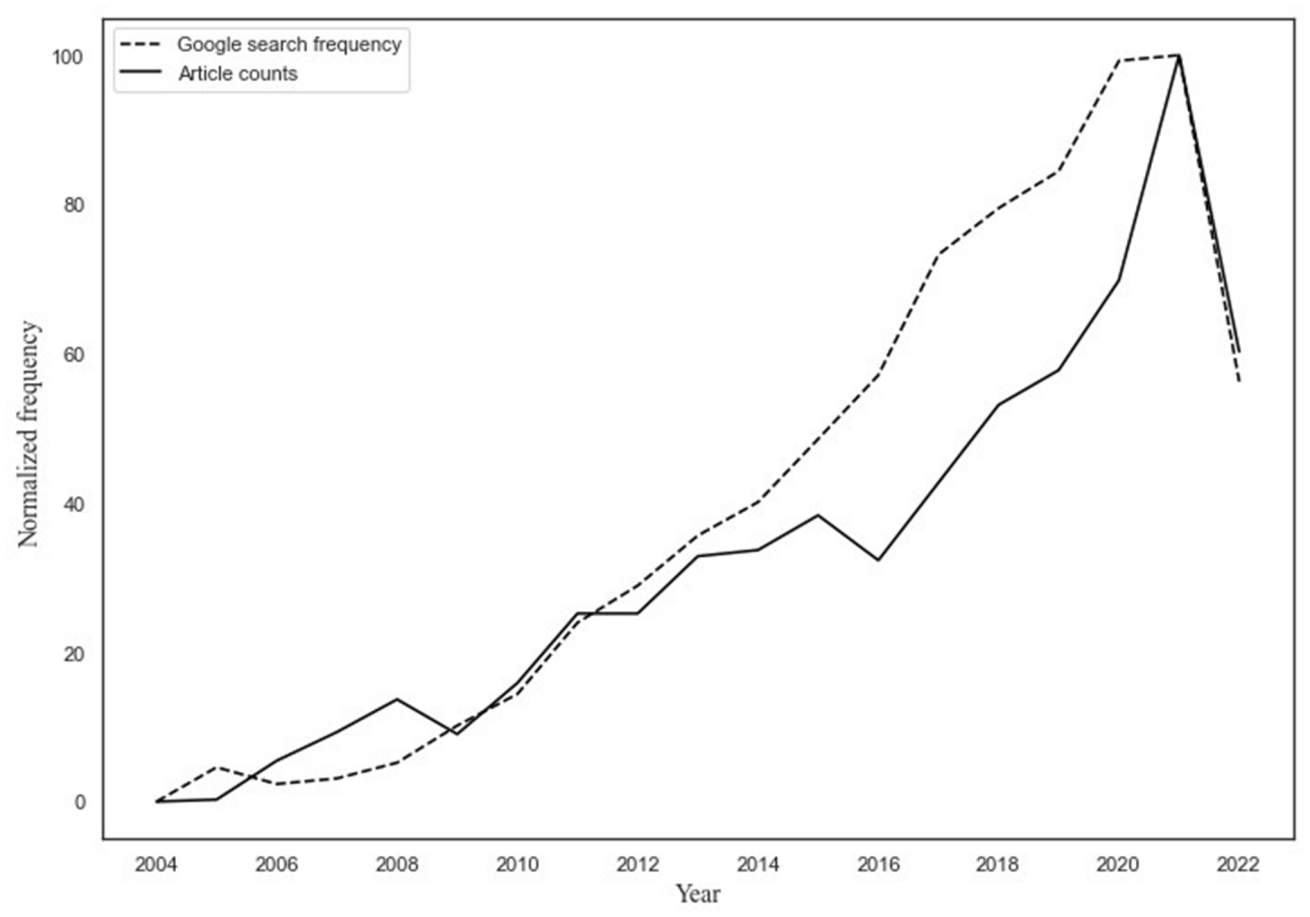
Depiction of how the frequency of published research articles on the sleep–pain relationship and the frequency of Google searches on sleep and pain follow each other over time. The *x*-axis depicts the time point (year), from 2004 to 2022, normalized to a scale ranging from 0 to 100. The *y*-axis depicts frequency of published articles or Google searches, normalized to a scale ranging from 0 to 100.

The number of published articles on the sleep–pain relationship per year, and the amount of Google searches per year (both scaled to 0–100) are displayed in Figure 3. As can be seen, the time trends closely follow each other from 2004 until 2022, and they both increase considerably over the course of the examined 17 years. The correlation between the article counts and the count of Google searches was strong and significant at *r* = 0.57. After controlling for autoregressive effects, the correlation remained significant and borderline moderate at *r* = 0.29. This indicates a close association between the two: that the increased interest in research on the sleep–pain relationship corresponds with an increased interest in sleep and pain among the general public.

### 3.5. The bidirectional, longitudinal relationship between research on the sleep–pain relationship and Google searches on sleep and pain

Table 1 depicts the results from the DSEM model with cross-lagged and autoregressive effects. When estimating the model, the Potential Scale Reduction Factor rapidly dropped to, and remained at, 1.000 across a range of Markov chain Monte Carlo iterations, up to 60,000. We found that more published articles predicted more future searches on Google (0.509, *p* < 0.001, 95% confidence interval (CI) [0.290, 0.860]), but not a future increase in published articles (0.103, *p* = 0.068, 95% CI [−0.035, 0.239]). More Google searches predicted a future increase in Google searches (0.976, *p* < 0.001, 95% CI [0.945, 1.001]), but not an increase in published research (0.011, *p* = 0.223, 95% CI [−0.018, 0.045]).

**Table 1:** Longitudinal, standardized Bayesian point estimates of frequency of published articles and Google searches in the dynamic structural equation model.

Effect	*b*	*p*	95% CI
Published articles → Future published articles	0.103	0.068	−0.035, 0.239
Google searches → Future Google searches	0.976	<0.001	0.945, 1.001
Published articles → Future Google searches	0.509	<0.001	0.290, 0.860
Google searches → Future published articles	0.011	0.223	−0.018, 0.045

The effects represent the aggregated effects of Time Point a on Time Point a + 1 across the measured time period, January 2004 to August 2022. CI, confidence interval.

## 4. Discussion

In this study, we first used a novel Big Data approach to assess how published research articles on the relationship between sleep and pain, and global Google searches on sleep and pain, have changed in frequency across time. In total, 4,357 articles on the sleep–pain relationship were identified, published from September 1896 to August 2022. The frequency of studies started to increase shortly after 1960 and then exponentially increased after the year 2000. The frequency of Google searches on sleep and pain, from January 2004 up until August 2022, steadily increased across the measured period. Second, we compared the time trends of published articles and Google searches using state-of-the-art statistical modeling: DSEM. The time trends significantly correlated with each other [*r* = 0.57] as well as when accounting for autoregressive effects [*r* = 0.29]. This indicates a close association between published research on the sleep–pain relationship and the general public’s interest in the same topic. It does not, however, give indications regarding the directionality of the relationship, and this is why we performed additional cross-lagged and autoregressive analyses.

We found that published research on the sleep–pain relationship leads to an increased interest in the sleep–pain relationship among the general public (0.509, *p* < 0.001, 95% CI [0.290, 0.860]). This is a blunt indicator that research on the sleep–pain relationship actually achieves a societal impact (Bornmann, 2012). Ideally, this will transfer into better clinical treatments, as well as self-help treatments for comorbid insomnia and chronic pain, in turn paving way for more effective prevention and treatment in the future. A theoretical model that may help explain this phenomena is the Framework of Practical Activity (Räsänen, 2015), which postulates that researchers need to be aware of their own practice of knowledge production in order to avoid creating a gap between theory and practice whereby the conceptual knowledge produced from research becomes disconnected by practitioners in the same field (Kallio and Houtbeckers, 2020). Because researchers who study the sleep–pain relationship generally are clinically oriented, it may be that they are successful in having their produced research resonate with both clinical practitioners and people who suffer from sleep and pain problems, a hypothesis that is in line with the current study’s results. We also found that the general public’s interest in the sleep–pain relationship reinforces itself over time (0.976, *p* < 0.001, 95% CI [0.945, 1.001]), and this was the strongest of the assessed effects. This result makes intuitive sense: It is well known that information flows through social contacts, which include both direct social contacts and communication across social media (Lewenstein, 1987; Bentley and Jyvik, 2010). It thereby makes sense that information about the sleep–pain relationship has spread among like-minded members of the general public.

We also found that neither published research nor the general public’s interest predicted more published research on the sleep–pain relationship. One possible explanation is that, although interest in the sleep–pain relationship is increasing rapidly, the topic may still be too specific to have an impact on the overall research community. If interest in the sleep–pain relationship continues to increase, however, this may change. The near-significant effect of published articles on future published articles (0.103, *p* = 0.068, 95% CI [−0.035, 0.239]) indicates a potential association. However, it may also be that researchers who study the sleep–pain relationship are good at communicating their research, but in the process of producing new knowledge they could be improve their listening to other sources, both regarding previous research and the general public. According to the Framework of Practical Activity, there is a danger in letting theoretical conceptualizations drive knowledge production: Relevant knowledge from clinical practitioners and people who suffer from sleep and pain problems may be ignored and lost and, over time, this preference for academic theory over practical experience may create a rift between academia and practice (Kallio and Houtbeckers, 2020). We therefore encourage researchers who study the sleep–pain relationship to be more apprehensive of—and inspired by—the general public’s discourse of the sleep–pain relationship moving forward, lest the promising carryover effect of published research on public interest cease over time.

This directional knowledge dissemination provides insights into health education and communication. As a well-established consensus (Meadows, 1987), accessible and understandable science knowledge is highly needed for society as a whole. For most health issues, equipping the public with knowledge involves making intellectual and public health contributions as a community-wide knowledge dissemination and is creating scalability for prevention. In a comparison of the style of writing between academic research and popular science articles, Bellés-Fortuño (2016) found that, even for undergraduate students, academic research information is difficult to process. Thus, popular science in the form of a wide range of media (e.g., podcasts, books, TV programs) fills an important gap between the academia and public. Moreover, the persuasive linguistic style often used in popular science writing can be more helpful in correcting/debugging politically infused health beliefs (e.g., conspiracy ideation about COVID-19 vaccines). To bridge these information discrepancies, scientists and medical practitioners should put more effort into disseminating easy-to-understand knowledge to the general public.

A major strength of the current study is the novel Big Data approach, whereby huge amounts of data were collected from two different types of sources, with the help of machine learning algorithms, and then merged together. Another strength is the use of novel and powerful statistical models, DSEM, which allowed us to analyze directionality in how published articles and the general public’s interest influenced each other. It also allowed us to control for autoregressive effects, which is a common source of bias in time-series data (Geiser, 2021). However, there are also limitations to the study that need to be addressed: We collected only scientific articles from Scopus and PubMed, potentially excluding articles that are registered only in other databases (especially non-English ones), as well as studies that were published. However, the included databases are among the largest available, and we believe that data from these suffice as a general indication of research interest. In addition, we trained our article-sorting algorithm on 1.5% of the total sample, which is lower than what Bannach-Brown et al. (2019) recommended. However, those recommendations were made for a systematic review, in which accuracy is of utmost importance. We believe our current training sample was sufficiently large for the purpose of the current study because its aim was to identify a general time trend. A third limitation was the blunt measure of the general public’s interest in sleep and pain; Google searches that included both “sleep” and “pain”; also, the API did not provide specific counts of searches. We recommend that future studies include more detailed and elaborate measures of the general public’s measures. At the same time, a strength with using Google searches is the almost unfathomably large and global sample of Google users from whom we gathered data.

Both academia and the general public show a rapidly increasing interest in the sleep–pain relationship, and research on this topic appears to bridge over into an interest among the general public. This is an encouraging and important finding because of the practical implications of sleep–pain research. Moving forward, however, we recommend that researchers who study the sleep–pain relationship be more wary of the general public’s knowledge of—and interest in—the sleep–pain relationship, to avoid potentially creating a rift between academic and practical knowledge.

## Data availability statement

The raw data supporting the conclusions of this article will be made available by the authors, without undue reservation.

## Author contributions

TA and XZ conceptualized the study. TA conducted the data collection and the statistical analyses. TA and XZ wrote the manuscript. All authors contributed to the article and approved the submitted version.

## Conflict of interest

The authors declare that the research was conducted in the absence of any commercial or financial relationships that could be construed as a potential conflict of interest.

## Publisher’s note

All claims expressed in this article are solely those of the authors and do not necessarily represent those of their affiliated organizations, or those of the publisher, the editors and the reviewers. Any product that may be evaluated in this article, or claim that may be made by its manufacturer, is not guaranteed or endorsed by the publisher.
