# Supplementary material for: Do both the research community and the general public share an interest in the sleep–pain relationship, and do they influence each other?
Source: Front Psychol. 2023 Jul 21;14:1198190. doi: 10.3389/fpsyg.2023.1198190 (PMC10400902; doi:10.3389/fpsyg.2023.1198190)
Supplement: Supplementary file 1 [file Data_Sheet_1.PDF]

Supplemental information

7.1. Python code to retrieve published articles and Google searches on the sleep-pain relationship.

```
pip install pybliometrics

%pip install pymed

import pandas as pd

import requests

import pybliometrics

import csv, re, json

import numpy as np

from datetime import datetime

from pybliometrics.scopus import config, AbstractRetrieval, ScopusSearch

from pybliometrics.scopus.utils import create_config

from pybliometrics.scopus.exception import Scopus404Error, Scopus429Error, Scopus500Error

from urllib.error import HTTPError

from urllib3.exceptions import ConnectionError, NewConnectionError

pybliometrics.scopus.utils.create_config()

APIKey = ['xxxxxxxxxx]

InstToken = ['xxxxxxx']

config["Authentication"]["APIKey"]= APIKey.pop()

config['Authentication']['InstToken']=InstToken.pop()

print(config['Authentication']['APIKey'])

print(config['Authentication']['InstToken'])

query = '( TITLE-ABS-KEY ( insomnia  OR  sleep )  AND  TITLE-ABS-KEY ( pain  OR  ache ) )
AND  ( LIMIT-TO ( LANGUAGE ,  "English" ) )'

scopus = ScopusSearch(query, refresh=True, view="STANDARD")

print("Number of records collected: {}.".format(scopus.get_results_size()))

list_eids_documents = scopus.get_eids()

def collect_data_manuscripts(list_eids_documents):

    data = []

    for key in list_eids_documents:

        record = {}
```

```
error = True
while error:
    try:
        paper = AbstractRetrieval(key, id_type="eid", view="FULL", refresh=True)
        error = False
        record["id"] = paper.identifier
        record["doi"] = paper.doi
        record["eid"] = paper.eid
        record["pii"] = paper.pii
        record["pubmed_id"] = paper.pubmed_id
        record["title"] = paper.title
        record["abstract"] = paper.abstract
        record["description"] = paper.description
        record["publication_date"] = datetime.strptime(paper.coverDate, "%Y-%m-%d").date() \
                            if paper.coverDate else None
        record["citation_num"] = paper.citedby_count
        record["language"] = paper.language
        record["production_type"] = paper.aggregationType
        record["source_type"] = paper.srctype
        record["auth_keywords"] = tuple(paper.authkeywords) if paper.authkeywords else None
        record["index_terms"] = tuple(paper.idxterms) if paper.idxterms else None
        record["issn"] = paper.issn
        try:
            record["isbn"] = " ".join(paper.isbn) if type(paper.isbn) == tuple else paper.isbn
        except TypeError:
            record["isbn"] = None
        record["conf_location"] = paper.conflocation
        record["conference_name"] = paper.confname
        record["vehicle_name"] = paper.publicationName
        record["vehicle_address"] = paper.publisheraddress
        record["title_edition"] = paper.issuetitle
        record["publisher"] = paper.publisher
```

```
record["affiliations"] = tuple(
    [{"id": affil.id if affil and affil.id else None,
      "affiliation": affil.name if affil and affil.name else None,
      "country": affil.country if affil and affil.country else None}
     for affil in paper.affiliation]) if paper.affiliation else None
record["subject_areas"] = tuple([area.area for area in paper.subject_areas]) \
                    if paper.subject_areas else None
record["authors"] = tuple(
    [{"id": author.auid if author and author.auid else None,
      "name": "{} {}".format(author.given_name, author.surname) \
            if author and author.given_name and author.surname else
          "{}".format(author.given_name) if author and author.given_name \
            and not author.surname else \
          "{}".format(author.surname) if author and author.surname \
            and not author.given_name else None}
     for author in paper.authors]) if paper.authors else None
record["author_affil"] = tuple(
    [{"id": author.auid if author and author.auid else None,
      "name": "{} {}".format(author.given_name, author.surname) \
            if author and author.given_name and author.surname else \
          "{}".format(author.given_name) if author and author.given_name \
            and not author.surname else \
          "{}".format(author.surname) if author and author.surname \
            and not author.given_name else None,
      "affil_id": author.affiliation_id if author and author.affiliation_id else None,
      "affiliation": author.organization if author and author.organization else None,
      "country": author.country if author and author.country else None}
     for author in paper.authorgroup]) if paper.authorgroup else None
record["ref_count"] = paper.refcount if paper.refcount else None
record["references"] = tuple([{"id": ref.id if ref and ref.id else None,
                    "title": ref.title if ref and ref.title else None,
                    "doi": ref.doi if ref and ref.doi else None,
```

```
                    "authors": ref.authors if ref and ref.authors else None}
                for ref in paper.references]) if paper.references else None
        except (Scopus404Error, Scopus500Error, HTTPError) as e:
            record["id"] = key
            print(key)
            error = False
        except Scopus429Error as e:
            config["Authentication"]["APIKey"] = _keys.pop()
            if len(_keys) == 0:
                raise e
        except (ConnectionError, NewConnectionError, Exception) as e:
            record["id"] = key
            print(key)
            error = False
      data.append(record)
   return data
Scopus_data = collect_data_manuscripts(list_eids_documents)
pd.DataFrame(Scopus_data).to_csv(r'C:\Users\toan\Desktop\Big Data\scopus_raw.csv', index=False,
quoting=csv.QUOTE_ALL)
Scopus_data.info()
from pymed import PubMed
pubmed = PubMed(tool="Search", email='tor.arnison@oru.se')
query_2 = "(pain OR ache) AND (insomnia OR sleep) AND (english[Language])"
num_max = pubmed.getTotalResultsCount(query_2)
print("Number of records to be returned: {}.".format(num_max))
results = pubmed.query(query_2, max_results=20873)
for article in results:
   print(type(article))
   print(article.toJSON())
articleList = []
articleInfo = []
for article in results:
   articleDict = article.toDict()
```

```
    articleList.append(articleDict)
for article in articleList:
    pubmedId = article['pubmed_id'].partition('\n')[0]
    articleInfo.append({u'pubmed_id':pubmedId,
                    u'title':article['title'],
                    u'abstract':article['abstract'],
                    u'doi':article['doi'],
                    u'keywords':keywords['keywords'],
                    u'publication_date':article['publication_date'],
                    u'authors':article['authors']})
articlesPD = pd.DataFrame.from_dict(articleInfo)
export_csv = pd.DataFrame(articlesPD).to_csv (r'C:/Users/toan/Desktop/Big Data/pubmed_raw2.csv', index = None, header=True)
print(articlesPD.head(10))
pd.DataFrame(results).to_csv(r'C:/Users/toan/Desktop/Big Data/results.csv', index=False, quoting=csv.QUOTE_ALL)
Pubmed = [paper.toDict() for paper in results]
Pubmed.head()
pd.DataFrame(Pubmed).to_csv(r"C:\Users\toan\Desktop\Big Data\pymed.csv", index=False, quoting=csv.QUOTE_ALL)
df_pymed = pd.read_csv(r"C:\Users\toan\Desktop\Big Data\pubmed_raw2.csv", header=0, dtype=object)
df_pymed.info()
pip install pylatexenc
import re, csv, pandas as pd, numpy as np
from pylatexenc.latex2text import LatexNodes2Text
from string import punctuation
def clean_title(title):
    if len(title) == 1 and title in punctuation:
        return None
    title = title.lower()
    title = title.replace("€", "").replace("…", "...").replace("τhe", "the").replace(
        "–", "-").replace("‘", "'").replace("“", "\"").replace("”", "\"").replace(
        "′", "'").replace("’", "'").replace("č", "c")
```

```
    while title[0] in punctuation or title[0] == " " or title[-1] in punctuation:
        if title[0] in punctuation:
            title = title[1:]
        if title[-1] in punctuation:
            title = title[:-1]
        title = title.strip()
    return re.sub(r"\"+", "", re.sub(r"\s+", " ", title))
df_scopusdata = pd.read_csv(r"C:\Users\toan\Desktop\Big Data\scopus_raw.csv", header=0,
dtype=object)
df_scopusdata.head(100)
df_scopusdata.info()
def clean_text(text, has_latex=False):
    if text:
        text = re.sub(r'\u2fff(s|\s)', r'\1', re.sub(r'\s+', ' ', re.sub(r"\ufeff\.?", "", re.sub(
            r"\\\\(\'\s)?", "", str(text))))).replace("\u200b", "").replace("\ue001", "").replace(
            "\ue061", "").replace("\u202f", "").replace("\u2060", "").replace("\u200f", "").replace(
            "\u200e", "").replace("\u202c", "").replace("–", "-").replace(
            "\u200c", "").replace("\\u0019", "").replace("\\s", "s").replace("\u202a", "").replace(
            "\u202d", "-").replace("\u0383", "-").replace("\u20f3", "ó").replace("\u20fa", "ú").replace(
            "\u2fff", "-").strip()
        text = text.replace("TNF-alpha induced", "TNF-α induced").replace(
            "TNF-Alpha induced", "TNF-α induced").replace("TNF- ⎵ induced", "TNF-α
induced").replace(
            "TNF-αinduced", "TNF-α induced").replace(
            "via NF- \u242c B pathway", "via NF-κB pathway").replace(
            "via NF-kappaB pathway", "via NF-κB pathway").strip()
     #  if has_latex:
     #      text = LatexNodes2Text().latex_to_text(re.sub("\\?%", "@PER@CENT@",
text)).replace("@PER@CENT@", "%")
      # text = re.sub(r"\s+", " ", re.sub(r"\-{2,}", "-", re.sub(r"\s?\xad(\s|\-)?", "-", text))).replace(
        #    "\\", "").replace("\\%", "%").replace("()", "").replace("[]", "").strip()
        return text
    else:
```

```
        return None
def clean_text(text, has_latex=False):
    if text:
        text = re.sub(r"\u2fff(s|\s)", r""\1", re.sub(r"\s+", " ", re.sub(r"\ufeff\.?", "", re.sub(
            r"\\\\(\'\s)?", "", str(text))))).replace("\u200b", "").replace("\ue001", "").replace(
            "\ue061", "").replace("\u202f", "").replace("\u2060", "").replace("\u200f", "").replace(
            "\u200e", "").replace("\u202c", "").replace("–", "-").replace(
            "\u200c", "").replace("\\u0019", "").replace("\\s", "s").replace("\u202a", "").replace(
            "\u202d", "-").replace("\u0383", "-").replace("\u20f3", "ó").replace("\u20fa", "ú").replace(
            "\u2fff", "-").strip()
        text = text.replace("TNF-alpha induced", "TNF-α induced").replace(
            "TNF-Alpha induced", "TNF-α induced").replace("TNF- ⎵ induced", "TNF-α
induced").replace(
            "TNF-αinduced", "TNF-α induced").replace(
            "via NF- \u242c B pathway", "via NF-κB pathway").replace(
            "via NF-kappaB pathway", "via NF-κB pathway").strip()
        # if has_latex:
        #     text = LatexNodes2Text().latex_to_text(re.sub("\\?%", "@PER@CENT@",
text)).replace("@PER@CENT@", "%")
        # text = re.sub(r"\s+", " ", re.sub(r"\-{2,}", "-", re.sub(r"\s?\xad(\s|\-)?", "-", text))).replace(
        #     "\\", "").replace("\\%", "%").replace("()", "").replace("[]", "").strip()
        return text
    else:
        return None
df_scopusdata = df_scopusdata.loc[df_scopusdata.id.notnull() & df_scopusdata.eid.notnull()]
df_scopusdata.replace({np.nan: None}, inplace=True)
df_scopusdata.citation_num.loc[df_scopusdata.citation_num.isnull()] = 0
df_scopusdata.ref_count.loc[df_scopusdata.ref_count.isnull()] = 0
df_scopusdata.abstract.loc[df_scopusdata.abstract.isnull() & df_scopusdata.description.notnull()] =
df_scopusdata.description.loc[
    df_scopusdata.abstract.isnull() & df_scopusdata.description.notnull()]
df_scopusdata.abstract.loc[df_scopusdata.abstract.notnull()] =
df_scopusdata.abstract.loc[df_scopusdata.abstract.notnull()].apply(
    lambda x: clean_text(x, True))
```

```
df_scopusdata.head(100)

df_scopusdata.vehicle_name.loc[df_scopusdata.conference_name.notnull() &
df_scopusdata.vehicle_name.notnull()] =
df_scopusdata.conference_name.loc[df_scopusdata.conference_name.notnull() &
df_scopusdata.vehicle_name.notnull()]

df_scopusdata.vehicle_name.loc[df_scopusdata.vehicle_name.notnull()] =
df_scopusdata.vehicle_name.loc[

    df_scopusdata.vehicle_name.notnull()].apply(clean_text)

df_scopusdata.title.loc[df_scopusdata.title.notnull()] =
df_scopusdata.title.loc[df_scopusdata.title.notnull()].apply(clean_text)

df_scopusdata.head()

columns_drop = ["eid", "pii", "description", "isbn", "conf_location", "conference_name",

    "vehicle_address", "title_edition"]

df_scopusdata.drop(axis=1, columns=columns_drop, inplace=True)

df_scopusdata.loc[:, ["citation_num", "ref_count"]] = df_scopusdata.loc[

    :, ["citation_num", "ref_count"]].astype(np.float32)

df_scopusdata.auth_keywords.loc[df_scopusdata.auth_keywords.notnull()] =
df_scopusdata.auth_keywords.loc[

    df_scopusdata.auth_keywords.notnull()].apply(eval)

df_scopusdata.index_terms.loc[df_scopusdata.index_terms.notnull()] =
df_scopusdata.index_terms.loc[

    df_scopusdata.index_terms.notnull()].apply(eval)

df_scopusdata.affiliations.loc[df_scopusdata.affiliations.notnull()] = df_scopusdata.affiliations.loc[

    df_scopusdata.affiliations.notnull()].apply(eval)

df_scopusdata.subject_areas.loc[df_scopusdata.subject_areas.notnull()] =
df_scopusdata.subject_areas.loc[

    df_scopusdata.subject_areas.notnull()].apply(eval)

df_scopusdata.authors.loc[df_scopusdata.authors.notnull()] =
df_scopusdata.authors.loc[df_scopusdata.authors.notnull()].apply(eval)

df_scopusdata.author_affil.loc[df_scopusdata.author_affil.notnull()] = df_scopusdata.author_affil.loc[

    df_scopusdata.author_affil.notnull()].apply(eval)

df_scopusdata.references.loc[df_scopusdata.references.notnull()] = df_scopusdata.references.loc[

    df_scopusdata.references.notnull()].apply(eval)

df_scopusdata.publication_date = pd.to_datetime(df_scopusdata.publication_date)

if "period" not in df_scopusdata:
```

```
    df_scopusdata["period"] = df_scopusdata.publication_date.apply(lambda x: "{}-{}".format(x.year,
x.month))
df_scopusdata.auth_keywords.loc[df_scopusdata.auth_keywords.notnull()] =
df_scopusdata.auth_keywords.loc[
    df_scopusdata.auth_keywords.notnull()].apply(lambda x: tuple([clean_text(item) for item in x]))
df_scopusdata.index_terms.loc[df_scopusdata.index_terms.notnull()] =
df_scopusdata.index_terms.loc[
    df_scopusdata.index_terms.notnull()].apply(lambda x: tuple([clean_text(item) for item in x]))
df_scopusdata.head()
for column in ["auth_keywords", "index_terms", "subject_areas"]:
    count = df_scopusdata.loc[df_scopusdata[column].notnull(), column][
                [np.any([item == None or item.lower() == "none" for item in items])
                 for items in df_scopusdata.loc[df_scopusdata[column].notnull(), column]]].size
    print("{}: {}".format(column, count))
for column in ["auth_keywords", "index_terms", "subject_areas"]:
    df_scopusdata.loc[df_scopusdata[column].notnull(), column] = [
        tuple([item for item in items if item])
        for items in df_scopusdata.loc[df_scopusdata[column].notnull(), column]]
    df_scopusdata.loc[df_scopusdata[column].notnull(), column] = df_scopusdata.loc[
        df_scopusdata[column].notnull(), column].apply(lambda x: x if len(x) > 0 else None)
df_scopusdata.authors.loc[df_scopusdata.authors.notnull()] = df_scopusdata.authors.loc[
    df_scopusdata.authors.notnull()].apply(lambda x: tuple(
        [{"id": item["id"], "name": clean_text(item["name"])} for item in x if item["id"]]))
for column in ["authors"]:
    df_scopusdata.loc[df_scopusdata[column].notnull(), column] = df_scopusdata.loc[
        df_scopusdata[column].notnull(), column].apply(lambda x: x if len(x) > 0 else None)
df_scopusdata.author_affil.loc[df_scopusdata.author_affil.notnull()] = df_scopusdata.author_affil.loc[
    df_scopusdata.author_affil.notnull()].apply(lambda x: tuple([{
        "id": item["id"] if item["id"] and item["name"] else \
            str(hash("{} - {}".format(item["name"], "Scopus"))) if item["name"] else None,
        "name": item["name"],
        "affil_id": item["affil_id"] if item["affil_id"] and item["affiliation"] else \
            str(hash("{} - {}".format(item["affiliation"], "Scopus"))) \
```

```
            if item["affiliation"] else None,
        "affiliation": item["affiliation"], "country": item["country"]}
    for item in x]))
df_scopusdata.author_affil.loc[df_scopusdata.author_affil.notnull()] = [
    set([(au["id"], au["name"], au["affil_id"],
        au["affiliation"], au["country"]) for au in row])
    for row in df_scopusdata.author_affil[df_scopusdata.author_affil.notnull()]]
df_scopusdata.author_affil.loc[df_scopusdata.author_affil.notnull()] = [tuple([dict(zip(
        ["id", "name", "affil_id", "affiliation", "country"], au)) for au in row])
    for row in df_scopusdata.author_affil[df_scopusdata.author_affil.notnull()]]
df_scopusdata = df_scopusdata.sort_values(by=["id", "period"]).drop_duplicates("id", keep="first")
df_scopusdata = pd.concat([df_scopusdata[df_scopusdata.title.isnull() | df_scopusdata.doi.isnull()],
    df_scopusdata[df_scopusdata.title.notnull() & df_scopusdata.doi.notnull()].sort_values(
        by=["title", "publication_date"]).drop_duplicates(
            ["title", "doi"], "last")], ignore_index=True)
df_scopusdata.references.loc[df_scopusdata.references.notnull()] = df_scopusdata.references.loc[
    df_scopusdata.references.notnull()].apply(lambda x: tuple(
        [{"id": ref["id"], "title": clean_text(ref["title"], True),
          "doi": clean_text(ref["doi"]), "authors": clean_text(ref["authors"], True)}
         for ref in x]))
def clean_id(text):
    if text:
        text = text.replace(".0", "").replace(
            "TNF-Alpha induced", "TNF-α induced").replace("TNF- ⎵ induced", "TNF-α
induced").replace(
            "TNF-αinduced", "TNF-α induced").replace(
            "via NF- \u242c B pathway", "via NF-κB pathway").replace(
            "via NF-kappaB pathway", "via NF-κB pathway").strip()
        return text
    else:
        return None
df_scopusdata.pubmed_id.loc[df_scopusdata.pubmed_id.notnull()] =
df_scopusdata.pubmed_id.loc[df_scopusdata.pubmed_id.notnull()].apply(clean_id)
```

```
df_scopusdata.head(100)

df_scopusdata.info()

df_scopusdata.to_csv(r"C:\Users\toan\Desktop\Big Data\scopus.csv", index=False,
quoting=csv.QUOTE_ALL)

df_scopus = pd.read_csv(r"C:\Users\toan\Desktop\Big Data\scopus.csv", header=0, dtype=object)

df_scopus.head()

df_scopus.info()

df_scopus.replace({np.nan: None}, inplace=True)

df_scopus.auth_keywords.loc[df_scopus.auth_keywords.notnull()] = df_scopus.auth_keywords.loc[
    df_scopus.auth_keywords.notnull()].apply(eval)

df_scopus.index_terms.loc[df_scopus.index_terms.notnull()] = df_scopus.index_terms.loc[
    df_scopus.index_terms.notnull()].apply(eval)

df_scopus.subject_areas.loc[df_scopus.subject_areas.notnull()] = df_scopus.subject_areas.loc[
    df_scopus.subject_areas.notnull()].apply(eval)

df_scopus.authors.loc[df_scopus.authors.notnull()] = df_scopus.authors.loc[
    df_scopus.authors.notnull()].apply(eval)

df_scopus.author_affil.loc[df_scopus.author_affil.notnull()] = df_scopus.author_affil.loc[
    df_scopus.author_affil.notnull()].apply(eval)

df_scopus.references.loc[df_scopus.references.notnull()] = df_scopus.references.loc[
    df_scopus.references.notnull()].apply(eval)

df_scopus.publication_date = pd.to_datetime(df_scopus.publication_date)

df_scopus["source"] = "Scopus"

df_scopus.title = df_scopus.title.apply(clean_title)

df_scopus.head()

df_scopus.info()

df_scopus.to_csv(r"C:\Users\toan\Desktop\Big Data\scopus.csv", index=False,
quoting=csv.QUOTE_ALL)

pip install pylatexenc

import re, csv, pandas as pd, numpy as np

from pylatexenc.latex2text import LatexNodes2Text

df_pubmeddata = pd.read_csv(r"C:\Users\toan\Desktop\Big Data\pubmed_raw2.csv", header=0,
dtype=object)

df_pubmeddata.info()
```

```
df_pubmeddata.info()
def clean_text(text):
    if text:
        return re.sub(r"\\", " ", re.sub(r"\s+", " ", re.sub(r"\-{2,}", "-", re.sub("[0-9]*\u200b", "",
            str(text)).replace("\xad", "-")).replace("\u2009", " ").replace("\xa0", " ").replace(
            "\n", " ").replace("\ufeff", "").replace("\u202f", "").replace("\u2028", " ").replace(
            "\u200f", "").replace("\u200e", "").replace("()", "").replace("[]", "").replace(
            "\\'", "\'").replace("\uf06b", "").replace("\x96", "").replace("\u200c", "")))).strip()
    else:
        return None
df_pubmeddata.replace({np.nan: None}, inplace=True)
columns_drop = ["methods", "conclusions", "results", "copyrights"]
df_pubmeddata.drop(axis=1, columns=columns_drop, inplace=True)
df_pubmeddata.pubmed_id = df_pubmeddata.pubmed_id.apply(lambda x: x.split()[0].strip())
df_pubmeddata.loc[df_pubmeddata.doi.notnull(), "doi"] =
df_pubmeddata.loc[df_pubmeddata.doi.notnull(), "doi"].apply(
    lambda x: x.split()[0].strip())
df_pubmeddata.info()
df_pubmeddata.keywords.loc[df_pubmeddata.keywords.notnull()] = [
    tuple([clean_text(keyword) for keyword in eval(keywords)]) if eval(keywords) else None
    for keywords in df_pubmeddata.keywords[df_pubmeddata.keywords.notnull()]]
df_pubmeddata[df_pubmeddata.keywords.notnull()].keywords[
    [np.any([item == None for item in keywords])
    for keywords in df_pubmeddata[df_pubmeddata.keywords.notnull()].keywords]].size
df_pubmeddata.keywords.loc[df_pubmeddata.keywords.notnull()] = [tuple([item for item in keywords
if item])
    for keywords in df_pubmeddata.keywords[df_pubmeddata.keywords.notnull()]]
df_pubmeddata.keywords.loc[df_pubmeddata.keywords.notnull()] = df_pubmeddata.keywords.loc[
    df_pubmeddata.keywords.notnull()].apply(lambda x: x if len(x) > 0 else None)
df_pubmeddata[df_pubmeddata.keywords.notnull()].keywords[
    [np.any([item == None for item in keywords])
    for keywords in df_pubmeddata[df_pubmeddata.keywords.notnull()].keywords]].size
for idx, authors in enumerate(df_pubmeddata.authors):
```

```
    if not eval(authors):
        df_pubmeddata.authors[idx] = None
    else:
        list_authors = []
        for author in eval(authors):
            auth = {}
            if author["firstname"] and author["lastname"]:
                auth["name"] = clean_text("{} {}".format(author["firstname"], author["lastname"]))
            elif author["firstname"] and not author["lastname"]:
                auth["name"] = clean_text(author["firstname"])
            elif not author["firstname"] and author["lastname"]:
                auth["name"] = clean_text(author["lastname"])
            else:
                auth["name"] = None
            auth["id"] = str(hash("{} - {}".format(auth["name"], "PubMed"))) if auth["name"] else None
            auth["affiliation"] = clean_text(author["affiliation"]) if "affiliation" in author else None
            auth["affil_id"] = str(hash("{} - {}".format(auth["affiliation"], "PubMed"))) \
                if auth["affiliation"] else None
            auth["country"] = None
            if auth["affiliation"] or auth["name"]:
                list_authors.append(auth)
        df_pubmeddata.authors[idx] = tuple(list_authors) if len(list_authors) > 0 else None
df_pubmeddata.rename(columns={"authors": "author_affil"}, inplace=True)
df_pubmeddata = pd.concat([df_pubmeddata[df_pubmeddata.title.isnull() |
df_pubmeddata.doi.isnull()],
    df_pubmeddata[df_pubmeddata.title.notnull() & df_pubmeddata.doi.notnull()].sort_values(
        by=["title", "publication_date"]).drop_duplicates(["title", "doi"], "last")], ignore_index=True)
df_pubmeddata['publication_date'] = pd.to_datetime(df_pubmeddata['publication_date'])
if "period" not in df_pubmeddata:
    df_pubmeddata["period"] = df_pubmeddata.publication_date.apply(lambda x: "{}-
{}".format(x.year, x.month))
df_pubmeddata.head()
df_pubmeddata.info()
```

```
df_pubmeddata.to_csv(r'C:\Users\toan\Desktop\Big Data\pubmed.csv', index=False,
quoting=csv.QUOTE_ALL)

df_pubmed = pd.read_csv(r'C:\Users\toan\Desktop\Big Data\pubmed.csv', header=0,
dtype={"pubmed_id": "str"})

df_pubmed.head()

df_pubmed.info()

df_pubmed.replace({np.nan: None}, inplace=True)

df_pubmed.author_affil.loc[df_pubmed.author_affil.notnull()] = df_pubmed.author_affil.loc[
    df_pubmed.author_affil.notnull()].apply(eval)

df_pubmed.publication_date = pd.to_datetime(df_pubmed.publication_date)

df_pubmed["source"] = "PubMed"

df_pubmed.title.loc[df_pubmed.title.notnull()] = df_pubmed.title.loc[
    df_pubmed.title.notnull()].apply(clean_title)

df_pubmed.head()

df_pubmed.info()

df_pubmeddata.to_csv(r'C:\Users\toan\Desktop\Big Data\pubmed.csv', index=False,
quoting=csv.QUOTE_ALL)

import re, csv, pandas as pd, numpy as np

from pylatexenc.latex2text import LatexNodes2Text

import pandas as pd

import requests

import pybliometrics

import csv, re, json

import numpy as np

from datetime import datetime

def clean_id(text):

    if text:

        text = text.replace(".0", "").strip()

        return text

    else:

        return None

df_pubmed = pd.read_csv(r'C:\Users\toan\Desktop\Big Data\pubmed.csv', header=0,
dtype={"pubmed_id": "str"})
```

```
df_pubmed.pubmed_id.loc[df_pubmed.pubmed_id.notnull()] =
df_pubmed.pubmed_id.loc[df_pubmed.pubmed_id.notnull()].apply(clean_id)

df_pubmed.info()

df_scopus = pd.read_csv(r"C:\Users\toan\Desktop\Big Data\scopus.csv", header=0, dtype=object)

df_scopus.info()

df_scopus.pubmed_id.loc[df_scopus.pubmed_id.notnull()] =
df_scopus.pubmed_id.loc[df_scopus.pubmed_id.notnull()].apply(clean_id)

df_pubmed.pubmed_id[df_pubmed.pubmed_id.isin(df_scopus.pubmed_id[df_scopus.pubmed_id.notn
ull()].values)].size

df_pubmed.pubmed_id[~df_pubmed.pubmed_id.isin(df_scopus.pubmed_id[df_scopus.pubmed_id.not
null()].values) &

  df_pubmed.title.isin(df_scopus.title.values) &

  df_pubmed.doi.isin(df_scopus.doi[df_scopus.doi.notnull()].values)].size

df_pubmed.loc[df_pubmed.pubmed_id.isin(df_scopus.pubmed_id.values) & df_pubmed.title.isnull(),
"title"] = \

  df_pubmed.pubmed_id[df_pubmed.pubmed_id.isin(df_scopus.pubmed_id.values) &
df_pubmed.title.isnull()].apply(

    lambda x: df_scopus.title[df_scopus.pubmed_id == x].iloc[0])

df_pubmed.loc[df_pubmed.pubmed_id.isin(df_scopus.pubmed_id.values) & df_pubmed.doi.isnull(),
"doi"] = \

  df_pubmed.pubmed_id[df_pubmed.pubmed_id.isin(df_scopus.pubmed_id.values) &
df_pubmed.doi.isnull()].apply(

    lambda x: np.reshape(df_scopus.doi[df_scopus.pubmed_id == x].values, -1)[0] \

      if df_scopus.doi[df_scopus.pubmed_id == x].size > 0 else None)

df_pubmed.loc[df_pubmed.doi[df_pubmed.doi.notnull()].isin(df_scopus.doi[df_scopus.doi.notnull()].
values) &

  df_pubmed.title.isnull(), "title"] = df_pubmed.doi[df_pubmed.doi[df_pubmed.doi.notnull()].isin(

    df_scopus.doi[df_scopus.doi.notnull()].values) & df_pubmed.title.isnull()].apply(

      lambda x: df_scopus.title[df_scopus.doi == x].item())

df_scopus.loc[df_scopus.pubmed_id.isin(df_pubmed.pubmed_id.values) & df_scopus.doi.isnull(),
"doi"] = \

  df_scopus.loc[df_scopus.pubmed_id.isin(df_pubmed.pubmed_id.values) & df_scopus.doi.isnull(),

    ["doi", "pubmed_id"]].apply(lambda x: df_pubmed.doi[df_pubmed.pubmed_id ==
x.pubmed_id].item() \

      if not x.doi else x.doi, axis=1)

df_scopus.loc[df_scopus.title.isin(df_pubmed.title[df_pubmed.title.notnull()].values)
```

```
    & df_scopus.pubmed_id.isnull() & df_scopus.doi[
        df_scopus.doi.notnull()].isin(df_pubmed.doi[df_pubmed.doi.notnull()].values), "pubmed_id"] = \
df_scopus.loc[df_scopus.title.isin(df_pubmed.title[df_pubmed.title.notnull()].values)
    & df_scopus.pubmed_id.isnull() & df_scopus.doi[
        df_scopus.doi.notnull()].isin(df_pubmed.doi[df_pubmed.doi.notnull()].values),
    ["pubmed_id", "title", "doi"]].apply(lambda x: x.pubmed_id if x.pubmed_id else np.reshape(
        df_pubmed.pubmed_id[(df_pubmed.title == x.title) & (df_pubmed.doi == x.doi)].values, -1)[0] \
            if df_pubmed.pubmed_id[(df_pubmed.title == x.title) & (df_pubmed.doi == x.doi)].size > 0 \
                else None, axis=1)
df_scopus.loc[df_scopus.pubmed_id.isin(df_pubmed.pubmed_id.values) & df_scopus.abstract.isnull(),
"abstract"] = \
    df_scopus.loc[df_scopus.pubmed_id.isin(df_pubmed.pubmed_id.values) &
df_scopus.abstract.isnull(),
        ["abstract", "pubmed_id"]].apply(lambda x: df_pubmed.abstract[
            df_pubmed.pubmed_id == x.pubmed_id].item()
                                    if not x.abstract else x.abstract, axis=1)
df_scopus.loc[~df_scopus.pubmed_id.isin(df_pubmed.pubmed_id.values)
             & df_scopus.title.isin(df_pubmed.title[df_pubmed.title.notnull()].values)
             & df_scopus.abstract.isnull()
             &
df_scopus.doi[df_scopus.doi.notnull()].isin(df_pubmed.doi[df_pubmed.doi.notnull()].values),
"abstract"] = \
df_scopus.loc[~df_scopus.pubmed_id.isin(df_pubmed.pubmed_id.values)
#              & df_scopus.title.isin(df_pubmed.title[df_pubmed.title.notnull()].values)
             & df_scopus.abstract.isnull()
             &
df_scopus.doi[df_scopus.doi.notnull()].isin(df_pubmed.doi[df_pubmed.doi.notnull()].values),
    ["abstract", "title", "doi"]].apply(lambda x: x.abstract if not x.abstract else np.reshape(
        df_pubmed.abstract[(df_pubmed.title == x.title)
             & (df_pubmed.doi == x.doi)].values, -1)[0] \
            if df_pubmed.abstract[(df_pubmed.title == x.title)
             & (df_pubmed.doi == x.doi)].size > 0 \
                else None, axis=1)
```

```
df_scopus.loc[df_scopus.pubmed_id.isin(df_pubmed.pubmed_id.values) &
df_scopus.auth_keywords.isnull(),
    "auth_keywords"] = df_scopus.loc[df_scopus.pubmed_id.isin(df_pubmed.pubmed_id.values) &
        df_scopus.auth_keywords.isnull(), ["auth_keywords", "pubmed_id"]].apply(
            lambda x: df_pubmed.auth_keywords[df_pubmed.pubmed_id == x.pubmed_id].item() \
                if not x.auth_keywords else x.auth_keywords, axis=1)
df_scopus.loc[~df_scopus.pubmed_id.isin(df_pubmed.pubmed_id.values) &
    df_scopus.title.isin(df_pubmed.title[df_pubmed.title.notnull()].values) &
df_scopus.auth_keywords.isnull() &
    df_scopus.doi[df_scopus.doi.notnull()].isin(df_pubmed.doi[df_pubmed.doi.notnull()].values),
"auth_keywords"] = \
df_scopus.loc[~df_scopus.pubmed_id.isin(df_pubmed.pubmed_id.values) &
    df_scopus.title.isin(df_pubmed.title[df_pubmed.title.notnull()].values) &
df_scopus.auth_keywords.isnull() &
    df_scopus.doi[df_scopus.doi.notnull()].isin(df_pubmed.doi[df_pubmed.doi.notnull()].values),
    ["auth_keywords", "title", "doi"]].apply(lambda x: x.auth_keywords if x.auth_keywords else
np.reshape(
        df_pubmed.auth_keywords[(df_pubmed.title == x.title) & (df_pubmed.doi == x.doi)].values, -
1)[0] \
            if df_pubmed.auth_keywords[(df_pubmed.title == x.title) & (df_pubmed.doi == x.doi)].size >
0 \
                else None, axis=1)
df_scopus.loc[df_scopus.pubmed_id.isin(df_pubmed.pubmed_id.values) &
df_scopus.author_affil.isnull(),
    "author_affil"] = df_scopus.loc[df_scopus.pubmed_id.isin(df_pubmed.pubmed_id.values) &
        df_scopus.author_affil.isnull(), ["author_affil", "pubmed_id"]].apply(
            lambda x: df_pubmed.author_affil[df_pubmed.pubmed_id == x.pubmed_id].item() \
                if not x.author_affil else x.author_affil, axis=1)
df_scopus.loc[df_scopus.pubmed_id.isin(df_pubmed.pubmed_id.values) &
df_scopus.subject_areas.isnull(),
    "subject_areas"] = df_scopus.loc[df_scopus.pubmed_id.isin(df_pubmed.pubmed_id.values) &
        df_scopus.subject_areas.isnull(), ["subject_areas", "pubmed_id"]].apply(
            lambda x: df_pubmed.subject_areas[df_pubmed.pubmed_id == x.pubmed_id].item() \
                if not x.subject_areas else x.subject_areas, axis=1)
idx_removed = df_pubmed.pubmed_id[df_pubmed.pubmed_id.isin(df_scopus.pubmed_id[
    df_scopus.pubmed_id.notnull()].values)].index.to_list()
```

```
idx_removed += df_pubmed.pubmed_id[~df_pubmed.pubmed_id.isin(df_scopus.pubmed_id[
        df_scopus.pubmed_id.notnull()].values) &
    df_pubmed.title.isin(df_scopus.title.values) &
    df_pubmed.doi.isin(df_scopus.doi[df_scopus.doi.notnull()].values)].index.to_list()
df_pubmed = df_pubmed[~df_pubmed.index.isin(list(set(idx_removed)))]
print("PubMed:", df_pubmed.pubmed_id.size)
print("Scopus:", df_scopus.id.size)
print("Expected total number of records for the final dataset:",
      (df_pubmed.pubmed_id.size + df_scopus.id.size))
df_final = pd.concat([df_pubmed, df_scopus], ignore_index=True)
df_final.replace({np.nan: None}, inplace=True)
df_final.rename(columns={"source": "data_source"}, inplace=True)
df_final.head()
df_final.info()
df_final.to_csv(r"C:\Users\toan\Desktop\Big Data\final.csv", index=False,
quoting=csv.QUOTE_ALL)
df_pubmed = pd.read_csv(r"C:\Users\toan\Desktop\Big Data\pubmed.csv", header=0, dtype=object)
df_scopus = pd.read_csv(r"C:\Users\toan\Desktop\Big Data\scopus.csv", header=0, dtype=object)
df_scopus.sample(300)
pd.options.display.max_rows = 300
pd.set_option("display.max_colwidth", None)
clean_dataframe = ['pubmed_id','doi','title','abstract','publication_date','period']
df_clean_scopus = df_scopus[clean_dataframe].copy()
df_clean_scopus.sample(300)
df_clean_pubmed.info()
df_train = pd.read_csv(r"C:\Users\toan\Desktop\Big Data\train_set_2.csv", header=0, dtype=object)
df_train.info()
df_final = pd.read_csv(r"C:\Users\toan\Desktop\Big Data\final.csv", header=0, dtype=object)
df_finaldup = df_final.drop_duplicates(subset=['pubmed_id'], keep='first', inplace=False,
ignore_index=False)
df_traindup = df_train.drop_duplicates(subset=['pubmed_id'], keep='first', inplace=False,
ignore_index=False)
df_finaldup.head()
```

```
df_data_train = pd.merge(df_finaldup, df_traindup, on="pubmed_id")
df_data_train.head(400)
df_data_train.info()
from sklearn.feature_extraction.text import CountVectorizer
vectorizer = CountVectorizer(lowercase   = True,
                         ngram_range = (1,1),
                         min_df      = 0,
                         max_df     = 1,
                         max_features = None)
vectorizer.fit(df_data_train['abstract'])
df_data_train.abstract = df_data_train.abstract.fillna(' ')
df_data_train.head()
df_data_train["text"] = df_data_train["title"] + [' '] + df_data_train["abstract"]
df_data_train.head(100)
vectorizer.fit(df_data_train['text'])
df_vectorized = vectorizer.transform(df_data_train['text'])
from sklearn.linear_model import LogisticRegression
lr_classifier = LogisticRegression(max_iter=1000)
lr_classifier.fit(df_vectorized, df_data_train['train'])
coefficients = pd.Series(lr_classifier.coef_[0],
                    index = vectorizer.get_feature_names())
coefficients.sort_values(ascending=False)[:15]
df_data_train['prediction']  = lr_classifier.predict(df_vectorized)
pd.crosstab(df_data_train['train'], df_data_train['prediction'])
from sklearn.metrics import classification_report, confusion_matrix, accuracy_score
accuracy_score(df_data_train['train'], df_data_train['prediction'])
print(classification_report(df_data_train['train'], df_data_train['prediction']))
from sklearn.model_selection import train_test_split
train, test = train_test_split(df_data_train, test_size=0.25)
len(train)
from sklearn.pipeline import Pipeline
pipeline = Pipeline([
```

```
                 ('vectorizer' , CountVectorizer()),
                 ('classifier' , LogisticRegression(max_iter=1000))
                ])

parameters = {'vectorizer__max_df' : [.7, .8, 0.9, 0.95, 0.99, 1],
            'vectorizer__min_df' : [1, 0.1, .01, .001, 0],
            'vectorizer__ngram_range' : [(1,1), (1,2), (1,3), (1,4), (2,2), (2,3), (3,3), (2,4), (3,4), (4,4)],
            }
from sklearn.model_selection import GridSearchCV
grid_search = GridSearchCV(pipeline,
                      parameters,
                      n_jobs = -1,
                      cv = 5,
                      verbose = 1)
grid_search.fit(train['text'],
             train['train'])
grid_search.best_score_
grid_search.best_estimator_
grid_search.best_params_
print(accuracy_score(train['train'],
                 grid_search.best_estimator_.predict(train['text'])))
print(accuracy_score(test['train'],
                 grid_search.best_estimator_.predict(test['text'])))
best_pipe = grid_search.best_estimator_
lr_prediction = best_pipe.predict(test['text'])
accuracy_score(test['train'],  lr_prediction)
print(classification_report(test['train'],lr_prediction))
df_final = pd.read_csv(r"C:\Users\toan\Desktop\Big Data\final.csv", header=0, dtype=object)
df_final.info()
df_final["text"] = df_final["title"] + ' ' + df_final["abstract"]
df_final.info()
clean_df = ['pubmed_id','doi','title','abstract','text','publication_date','period']
```

```
df_final_clean = df_final[clean_df].copy()

df_final_clean.head()

df_final_clean.info()

df_final_clean.to_csv(r"C:\Users\toan\Desktop\Big Data\final_clean.csv", index=False,
quoting=csv.QUOTE_ALL)

df_final_clean.text = df_final_clean.text.fillna(' ')

df_final_clean['prediction']  = best_pipe.predict(df_final_clean['text'])

df_final_clean.head()

df_final_clean['prediction'].value_counts()

df_sleeppain = df_final_clean.loc[df_final_clean['prediction'] == '1']

df_sleeppain.info()

df_sleeppain.to_csv(r"C:\Users\toan\Desktop\Big Data\sleeppain.csv", index=False,
quoting=csv.QUOTE_ALL)

df_sleeppain = pd.read_csv(r"C:\Users\toan\Desktop\Big Data\sleeppain.csv", header=0,
dtype=object)

df_sleeppain.info()

df_sleeppain['counts'] = df_sleeppain['period'].map(df_sleeppain['period'].value_counts())

df_sleeppain.head(100)

df_columns = ['period', 'counts']

df_articlecount = df_sleeppain[df_columns].copy()

df_articlecount.info()

df_articlecount.head(100)

df_articlecount = df_articlecount.dropna(subset = ['period'])

df_articlecount.info()

pd.set_option('display.max_rows', None)

df_articlecount.head(1000)

df_articlecount = df_articlecount.sort_values( by='period', ascending=True)

df_articlecount.head(5000)

df_articlecount = df_articlecount.drop_duplicates(subset='period', keep='first', inplace=False,
ignore_index=False)

df_articlecount.head(5000)


df_articlecount.to_csv(r'C:\Users\toan\Desktop\Big Data\articles.csv')

! Pip install pytrends
```

```
from pytrends.request import TrendReq

pytrends = TrendReq(hl = 'en-US', tz = 360)

kw_list = ['sleep pain']

pytrends.build_payload(kw_list, timeframe='all')

df_google = pytrends.interest_over_time()

df_google

df_google.to_csv(r'C:\Users\toan\Desktop\Big Data\google.csv')

df_articles = pd.read_csv(r"C:\Users\toan\Desktop\Big Data\articles_2.csv", header=0, dtype=object)

df_articles = df_articles.drop(['Unnamed: 0'], axis=1)

df_articles = df_articles.sort_values(by='period')

df_articles['period'] = pd.to_datetime(df_articles['period'], format = '%Y-%m')

df_articles = df_articles.sort_values(by='period')

df_articles.tail(1000)

df_google = pd.read_csv(r"C:\Users\toan\Desktop\Big Data\google_2.csv", header=0, dtype=object)

df_google = df_google.rename(columns={'date': 'period'})

df_google = df_google.drop(['isPartial'], axis=1)

df_google['period'] = pd.to_datetime(df_google['period'], format = '%Y-%m')

df_google = df_google.sort_values(by='period')

df_articles.head(250)

ts_data = pd.merge(df_google, df_articles, on="period")

ts_data.head(250)

ts_data.to_csv(r'C:\Users\toan\Desktop\Big Data\mplus.csv')

ts_data['counts'] = ts_data['counts'].astype(int)

ts_data['counts'] = ((ts_data['counts'] - ts_data['counts'].min()) / (ts_data['counts'].max() -
ts_data['counts'].min()) * 100)

display(ts_data)

ts_data = ts_data.rename(columns={'counts': 'Article counts', 'sleep pain': 'Google searches', 'period':
'Month'})

ts_data.head(500)

ts_data.to_csv(r"C:\Users\toan\Desktop\Big Data\ts_data.csv", index=False,
quoting=csv.QUOTE_ALL)

pip install matplotlib

import seaborn as sns
```

```
df_articles['counts'] = df_articles['counts'].astype(int)

df_articles['period'] = pd.to_datetime(df_articles['period'], format = '%Y-%m')

df_articles = df_articles.resample('Y', on='period').sum()

df_articles = df_articles.reset_index()

df_articles.head(100)

from matplotlib import rcParams

rcParams['figure.figsize'] = 11.7,8.27

df_articles = df_articles.rename(columns={'counts': 'Article counts', 'period': 'Year'})

sns.lineplot(data=df_articles, x="Year", y="Article counts")

df_articles.tail(100)

df_google = pd.read_csv(r"C:\Users\toan\Desktop\Big Data\google_2.csv", header=0, dtype=object)

df_google = df_google.rename(columns={'date': 'period'})

df_google = df_google.drop(['isPartial'], axis=1)

df_google.head()

df_google['period'] = pd.to_datetime(df_google['period'], format = '%Y-%m')

df_google['sleep pain'] = df_google['sleep pain'].astype(int)

df_google = df_google.resample('Y', on='period').sum()

df_google = df_google.reset_index()

df_google.head(20)

df_google = df_google.rename(columns={'sleep pain': 'Google searches', 'period': 'Year'})

df_google['Google searches'] = df_google['Google searches'].astype(int)

df_google['Google searches'] = ((df_google['Google searches'] - df_google['Google searches'].min()) /
(df_google['Google searches'].max() - df_google['Google searches'].min()) * 100)

df_google.head(100)

sns.lineplot(data=df_google, x="Year", y="Google searches")

import pandas as pd

import requests

import pybliometrics

import csv, re, json

import numpy as np

from datetime import datetime

ts_data = pd.read_csv(r"C:\Users\toan\Desktop\Big Data\ts_data.csv", header=0, dtype=object)

ts_data.info()
```

```
ts_data['Article counts'] = ts_data['Article counts'].astype(float)

ts_data['Article counts'] = ts_data['Article counts'].round(decimals=0)

ts_data.info()

ts_data.tail(100)

ts_data['Article counts'] = ts_data['Article counts'].astype(int)

ts_data['Google searches'] = ts_data['Google searches'].astype(int)

ts_data['Month'] = pd.to_datetime(ts_data['Month'], format = '%Y-%m')

ts_data = ts_data.resample('Y', on='Month').sum()

ts_data.head(20)

ts_data = ts_data.reset_index()

ts_data.head()

ts_data['Article counts'] = ((ts_data['Article counts'] - ts_data['Article counts'].min()) / (ts_data['Article
counts'].max() - ts_data['Article counts'].min()) * 100)

ts_data['Google searches'] = ((ts_data['Google searches'] - ts_data['Google searches'].min()) /
(ts_data['Google searches'].max() - ts_data['Google searches'].min()) * 100)

ts_data.head(20)

ts_data = ts_data.rename(columns={'Month': 'Year'})

ax = ts_data.plot(x="Year", y="Google searches", legend=False)

ax2 = ax.twinx()

ts_data.plot(x="Year", y="Article counts", ax=ax2, legend=False, color="r")

ax.figure.legend()

ts_data.to_csv(r'C:\Users\toan\Desktop\Big Data\ts_data_y.csv')

correlation = ts_data.corr()

print(correlation)
```

7.2. Mplus syntax to analyze the time-series data using DSEM.

```
DATA:
File = 'C:\Users\toan\Desktop\Big data\mplus_3.txt';
VARIABLE:
Names = time date google article;
Usevariables = google article;
Lagged = google (1) article (1);
!Missing = All (*insert*);
ANALYSIS:
Estimator = Bayes;
Processors = 2;
Biterations = (49999);
fbiterations = 60000;
!thin = 50;
MODEL:
google on google&1;
article on article&1;
google on article&1;
article on google&1;
OUTPUT:
tech8 standardized tech4 residual
PLOT:
Type = plot3;
```
